# Historicising “containment and delay”: COVID-19, the NHS and high-risk patients

**DOI:** 10.12688/wellcomeopenres.15962.1

**Published:** 2020-06-10

**Authors:** Martin D. Moore

**Affiliations:** 1 Wellcome Centre for Cultures and Environments of Health, University of Exeter, Exeter, EX4 4QH, UK

**Keywords:** pandemics, Charles Rosenberg, National Health Service, risk, general practice, health inequalities

## Abstract

Despite the first case of the novel coronavirus only being reported to the WHO at the end of December 2019, humanities and social science scholars have been quick to subject local, national and international responses to COVID-19 to critique. Through television and radio, blogs, social media and other outlets, historians in particular have situated the ongoing outbreak in relation to previous epidemics and historicised cultural and political responses. This paper furthers these historical considerations of the current pandemic by examining the way the National Health Service (NHS) and discourses of risk have figured in public and policy responses. It suggests that appeals to protect the NHS are based on longer-term anxieties about the service’s capacity to care and endure in the face of growing demand, as well as building on the attachment that has developed as a result of this persistence in the face of existential threats. Similarly, the position of elderly, vulnerable and “at risk” patients relates to complex histories in which their place in social and medical hierarchies have been ambiguous. It thus argues that the ways in which time appears as both a threat and a possibility of management in the current crisis form part of a longer trajectory of political and cultural thinking.

## COVID-19, the NHS and high-risk patients

Historians and social scientists have long theorised the ‘sudden disastrous event’ of epidemics as social and cultural stress tests ( Porter, 1999, p. 79). In a classic exploration of the AIDS crisis in the late 1980s, Charles Rosenberg conceptualised the temporal and ‘dramaturgic form’ of epidemics, noting that they mobilised ‘communities to act out proprietory [sic] rituals that incorporate and reaffirm fundamental social values and modes of understanding’. Together with their ‘unity of place and time’, this public character meant that – for scholars – epidemics formed ‘an extraordinarily useful sampling device – at once found objects and natural experiments capable of illuminating fundamental patterns of social value and institutional practice’ as well as widely-shared ‘cultural assumptions’ ( Rosenberg, 1989, p. 2).

Despite the first case of the novel coronavirus only being reported to the WHO at the end of December 2019, humanities and social science scholars have been quick to subject local, national and international responses to COVID-19 to critique ( Manderson & Levine, 2020) – some even applying Rosenberg’s dramaturgy in their analyses. Through television and radio, blogs, social media and other outlets, historians in particular have situated the ongoing outbreak in relation to previous epidemics (most notably the 1918 flu pandemic), and historicised cultural and political responses – especially that of quarantine (though cf: Lachenal & Thomas, 2020). Indeed, the peculiarity of the UK government’s own measures have also been historicised in relation to its political, economic and public health histories.

However, further consideration of the way the National Health Service (NHS) and discourses of risk have figured in public and policy responses to COVID-19 can also reveal the way in which historical precedents are continuing to shape contemporary life in relation to the epidemic. Appeals to protect the NHS are based on longer-term anxieties about the service’s capacity to care and endure in the face of growing demand, as well as building on the attachment that has developed as a result of this persistence in the face of existential threats. Similarly, the position of elderly, vulnerable and “at risk” patients relates to complex histories in which their place in social and medical hierarchies have been ambiguous. The ways in which time appears as both a threat (too much demand, too little time to cope) and a possibility of management (delay attending, target bodies with better chances of survival and utility) in the current crisis form part of a longer trajectory of political and cultural thinking.

## The NHS in “Contain and Delay”

As the mass celebrations of the National Health Service’s 70 ^th^ “anniversary” in 2018 attested, the British public has developed a particularly strong psychosocial attachment to the NHS ( BBC Four, 2018); following Rosenberg, its centrality to practices of governance and social and cultural configurations during the ongoing COVID-19 epidemic is indeed telling. A core, and very visible, feature of the UK’s “contain and delay” strategy has been to appeal to the public to, in essence, stay away from institutions of the NHS as much as possible. Ministerial podia have been adorned with slogans, repeated by the Prime Minister and Public Health England, to ‘stay home, protect the NHS, save lives’ (see also: Baraitser & Salisbury, 2020, *Waiting in Pandemic Times*). On one level, there is a very practical aim in this appeal, as implied within the Department of Health and Social Care’s policy papers: this distance will prevent overwhelming services and ensure that spaces associated with other forms of medical containment and delay are not themselves sites for spreading the virus among the public and vulnerable key workers. As with previous, smaller outbreaks of conditions like Swine Flu in 2009, delaying the spike in cases until the summer will have benefits as ‘flu and other winter bugs are not driving GP consultations and hospital admissions’ ( Department of Health and Social Care, 2020). In short, the public’s failure to wait for care of other conditions might not only threaten individual lives, but the life of the service itself.

At the same time, the appeal to ‘protect the NHS’ also aims to leverage cultural love and attachment to the service in order to encourage adherence to social distancing regulations. This has not only been seen in the way that “protecting” the Service and “saving lives” literally follow on from “staying home” in the grammatical construction of the soundbite; NHS workers themselves have posted signs on social media attesting that ‘we stay here for you – please stay home for us’. Such appeals construct the service as a subject that waits for citizens in their time of need (though cf: Davies, 2020, *Waiting in Pandemic Times*), and uses this temporal dedication as a way to suggest that the social rights of citizenship come with expectations of performing health-protective behaviours ( Berridge, 2007; Mold *et al.*, 2019; Reubi & Mold, 2013). Appreciation for the risk health workers experience in waiting for, and with, our infected selves has been manifest in overt displays of ‘clapping for carers’. The designation of “carer” nominally broadens this appreciation from medical professionals to encompass everyone involved in forms of care work. However, though for some this broad appellation holds (and in spite of the international origins of the practice), association with the National Health Service in particular is evident in the use of additional media, notably signs saying “thank you NHS”.

## Historicising NHS attachment and its existential threats

Returning to Rosenberg’s framing, however, we might consider these developments in a more historical light. In his first exploration of epidemics as a ‘sampling technique’, Rosenberg used the example of cholera epidemics in America during 1832, 1849 and 1866 to detail and explain ‘the magnitude of the changes effected in American society’ between those years ( Rosenberg, 1962 [1987], p. 4). The changing responses to epidemics in those years highlighted in particular the effects of secularisation, urbanisation, and a growing materialism and rationalism on public health. By contrast, political, societal, and public health responses to COVID-19 have shown up how a number of long-term discourses and practices that still structure health governance.

For instance, the current cultural expression of attachment to the NHS is in some ways at a peak in the present, most likely as a result of post-imperial globalisation, austerity and a broad remaking of the welfare state since 2010 (on austerity’s ongoing effects: Osserman & Lê, 2020, *Waiting in Pandemic Times*). Large sections of the British public configured post-2008 financial stringency and large-scale structural change as an existential threat to the NHS, reacting to these developments with growing campaigns to “save our NHS”. Failures to meet prominent performance metrics, most tellingly waiting times ( Sheard, 2018), were put forward as evidence of mortal under-investment (rather than offering a sign of poor care), and so powerful has this response been that post-Brexit politics have been fought on the battleground of who would spend the most on the health service. Indeed, the fact that the NHS simultaneously assumed centre stage in the consciously inclusive Olympics opening ceremony and the divisive, and consciously exclusionary, Brexit campaign, is indicative of how the service has been integrated into diverse visions of post-imperial British identity in ways impossible to foresee in 1948. As leading historians of the service have suggested, moreover, the sheer media saturation of the most recent “anniversary” was on a scale not seen in previous reflections ( Bivins *et al.*, 2018).

However, this appreciation of the service is certainly not novel. One Mass Observation participant in 1949 remarked how the NHS was ‘one of the finest things that ever happened in this country’, and a ‘godsend’ for people previously priced-out from healthcare under earlier mixed economies of care, even using waiting itself – the ‘crowded doctor’s surgeries and queues for spectacles’ – as evidence ( Mass Observation, 1949). Likewise, letters to the press shortly after the tenth year of operation noted how any ‘proposed survey will show that the ten years of the NHS, in spite of many difficulties and mistakes inevitable in a new social experiment, has done much’, most notably extending ‘the provision of medical services to all, without payment at the time of need’ ( Barrow, 1959). For these commentators, an appreciation of the NHS was born from direct experience of the painful social exclusions of mixed systems of provision; a combination of state insurance, mutual funds, contributory schemes, public assistance and private procurement that – though expanding interwar healthcare coverage considerably ( Doyle, 2014) – had failed to provide rights of access to many who were unemployed or “dependent” (such as married women and children), and which was regularly criticised for its inequalities and inequities ( Digby, 1999, pp. 306–24; Gorsky, 2011a). Yet, even as time passed, new generations were born, and these experiences moved more to the margins of living memory, attachment to the service did not fade. Foreshadowing current developments, existential threats to the NHS’s capacity to wait for its ill subjects mobilised populations in its defence. Activism around hospital closures locally in the 1960s and 1970s transformed over the 1980s into a defence of the NHS nationally ( Crane, 2019). At first, growing affect for the NHS emerged as the public interacted with its local institutions ( Crane & Hand, *unpublished study*), before becoming the focus of left-wing political resistance to Thatcherite reforms and grounds for an identification with the values of universality and equity within the service ( Crane, 2019). Into the 1990s and 2000s, moreover, this attachment also became a focus for more overt political management, with the development of national logos and Prime Ministerial forewords to NHS histories (see, for instance, Tony Blair’s inscription for Rivett, 1998).

Equally, current-day constructions of COVID-19 as a hazard that could overwhelm the NHS’s capacity to endure have precedents. Medical professionals warned the public about overwhelming the NHS almost as soon as it launched. Early forecasts for the cost of the NHS to the Treasury were predicated on problematic assumptions ( Cutler, 2003) – itself highlighting the difficulties of modelling the future that have haunted discussions of COVID-19 ( Hinchliffe, 2020). As Roberta Bivins has noted, the ‘advent of the NHS, with its promise of free access to a complete medical service, released a tidal wave of pent-up medical need, and shone a spotlight on the complete inadequacy of existing systems to meet that need’ ( Bivins, 2015, p. 12). Local medical authorities discussed the possibility of a ‘breakdown of the hospital system’ ( Exeter & Mid-Devon Hospital Management Committee, 1950, p. 102), and newspapers ran headlines of ‘grave situation’ within Britain’s hospitals ( Exeter Express & Echo, 1950).

General Practitioners (GPs) were perhaps most sensitive to this surge in demand, however. Partly this was because they acted as the gatekeepers to hospitals. They were thus the first port of call for all problems that patients felt required medical assistance ( Loudon & Drury, 1998). Yet, their complaints about growing workload were also driven by the politics of a largely conservative profession. The British Medical Association (BMA) had fought strongly against a universal health service, and many GP members expressed anxieties about a loss of independence should salaried service be imposed ( Klein, 2006). Despite retaining their position as independently contracted workers after 1948 ( Lewis, 1998), GPs regularly lamented their loss of status relative to the patient, complaining that the “freeness” of the NHS removed any economic or psychological barrier for patients to attend surgeries. For some GPs, the NHS was thus most notable for the ‘changed attitude of the patient – the demanding attitude’ it produced ( British Medical Journal, 1949, p. 199), with a minority going so far as to complain that patients treated them as ‘a servant’ ( Hadfield, 1953, p. 699). For others, without the fee, patients filled up their waiting rooms with trivial complaints ( Cartwright, 1967, pp. 44–52), as ‘the old pride in not going to the doctor unless it was absolutely necessary’ disappeared ( Weir, 1953, p. 2).

Regardless of the service’s continued existence (and popularity), GPs warned that the NHS was strained to its financial limit, alleging that supposed patient greed and short-sightedness risked the whole enterprise. ‘People used not to attend the doctor for colds or a nose-bleed before 1948’, lamented one practitioner. ‘Now more than half the patients in the average doctor’s waiting room have no right to be there. They are sabotaging the service and stealing their own money’. The letter continued to appeal to patients to recognise that ‘doctors are human and have only limited powers of endurance’ and, though not formally asking them to stay away from the service, offered suggestions for interacting with the GP, such as leaving messages at the right time and avoiding night calls ‘if you can’ ( G.P., 1951, p. 2.). Into the 1960s, the BMA produced posters asking patients to ‘help your doctor to help you’ through their behaviour ( British Medical Journal, 1962, p. 4) – a campaign that earned Ministry of Health approval ( British Medical Journal, 1966) – whilst Conservative politicians argued that waiting rooms filled with ‘more and more people whose only complaint is that they are refusing to pay for their own aspirins and cotton-wool’ risked the service’s operation by reducing GP recruitment and retainment ( British Medical Journal, 1965, p. 1317). By the 1970s, some GPs had even argued that ‘the financial survival of the NHS probably depends to quite a large extent’ on patients’ capacity to wait, abstain, and endure outside of the service, to treat their own “minor ailments” before seeking consultation ( Marsh, 1978).

These concerns dovetailed with broader efforts among GPs to limit the temporal extent of their duties. GPs pointed to the physical and mental strain of their 24-hour a day, 7-day a week contracts, suggesting their life was one of ‘constant anxiety’ as a result of this ‘continuing responsibility’ ( Manchester Guardian, 1958). In response, over the 1960s, 1970s and 1980s, they produced appointment systems, rotas, out of hours services, and demanded holiday as a way to curb the effects on their social and psychological life ( Armstrong, 1985). Political agreements and financial arrangements struck in the 1966 GP Charter facilitated such innovative modes of time reclamation ( Bosanquet & Salisbury, 1998; Lewis, 1998). However, such complaints and innovations also neatly aligned with the longer-term politics of service funding, as well as efforts to ensure patients sought the right attention for their particular ills. The financing of the service has been the focus of consistent dispute since its foundation, but since the 1980s healthcare professionals, left-wing politicians and critics of service retrenchment have mobilised models of health service specific inflation to critique existing levels of investment ( Klein, 2006, pp. 142–6). Likewise, one only need peruse the range of posters created to help patients “choose” the appropriate service for their complaints to see the way that concerns about patient decision-making was problematised in relation to financial constraints into the twenty-first century (see also, NHS England’s own ‘Time to Care’ initiatives: Davies, 2020).

## Ambiguities of risk and vulnerability

These efforts to target services for particular type of patients in a bid to reduce money had been foreshadowed by programmes to re-site chronic disease care from hospitals to general practice during the 1970s and 1980s ( Moore, 2019). Their languages and practices of risk management – spreading out from post-war epidemiology ( Berlivet, 2005; Oppenheimer, 2006; with precedent in early twentieth century medical insurance: Rothstein, 2003) – have also found echoes in efforts to deal with COVID-19. People considered particularly vulnerable to the virus on the basis other health conditions have been categorised as ‘very high risk’ and advised to self-isolate for a considerably longer period than the general population. This emphasis on prioritisation has manifested in other social measures – such as attempts to provide preference in home food delivery or reserved shopping times – encompassing other groups considered vulnerable, like the over 70s. Building on the changing tone of public health campaigns from the 1950s onwards, efforts to control individual behaviour have also looked to mobilise emotional responses to the risks to these groups ( Berridge & Loughlin, 2005; Elizabeth *et al.*, 2019; Hand, 2020). The language of self-isolation – as opposed to quarantine – not only highlights the individual’s responsibility in the crisis, but “staying home” has been framed as something which will also save the lives of the most vulnerable – our parents, grandparents or sick relatives.

At the same time, political and media responses have also underlined the marginal status of the most vulnerable groups. Many early reports of COVID-19 deaths came with claims that the patient was either old or had ‘underlying health conditions’. These appeals were almost intended as reassurances, a call to reduce the alarm or panic of the supposedly young and fit. Once again, such strategies recall the prioritisation of clinical and public health services towards those who might be considered productive or reproductive, and were seen to be reproductive of particular national subjects. Though the political, cultural and economic factors driving development were complex, the growth of public British health services during the twentieth century nonetheless began with national insurance tied to employment ( Gorsky, 2011b) and antenatal, maternal and child welfare services, which developed within contexts of eugenic, imperial and racist discourses of ‘racial fitness’ ( Porter, 1999, pp. 165–95). At the same time, over the late nineteenth and early twentieth centuries hospitals frequently tried to exclude “chronic” and elderly patients from their walls, resulting in their institutionalisation in old poor law hospitals with little emphasis on rehabilitation or care ( Levene, 2009; Weisz, 2014).

Even with the creation of the NHS, the language of inclusion for elderly patients and people with long-term illness or diverse physical impairments was often at odds with practice. For instance, geriatrics and rehabilitation specialisms developed as means to prevent “bed blocking” in acute hospitals, and they received little state support ( Bridgen, 2001; Gorsky, 2013; Martin, 1995; Thane, 2003). Elderly patients found themselves stuck between divisions of health and social services, with neither wanting (nor having the budgets) to provide the support and care required ( Bridgen & Lewis, 1999, though also: Welshman, 1996). Indeed, the marginality of age, impairment and long-term illness intersected with structural discrimination and the politics of race and migration. Since its foundation in 1948, the NHS has depended upon – and been shaped by – racialised and migrant labour ( Kyriakides & Virdee, 2003; Simpson, 2018), with many of these ‘architects’ of the service now aging and dying within its walls ( Gunaratnam, 2013). Xenophobia in the medical professional meant that “marginal” specialties like geriatrics were developed by migrant doctors ( Bornat *et al.*, 2016). Despite its reliance on such a diverse labour force, however, the health services nonetheless vacillated between hostility and violent indifference towards racialised patients ( Bivins, 2015), particularly those with chronic conditions ( Ahmad, 2000). The NHS was even incorporated into hostile environment policies that wreaked further damage to these patients, NHS staff, their families and communities ( Gunaratnam, 2013). In the absence of concerted state efforts to address inequalities, the needs of – and support and services for – elderly, “disabled”, chronically ill and racialised patients thus became the focus of political activism and charity, as well as local co-operation with interested clinicians and service providers ( Bivins, 2007; Jackson, 2009, p. 21; Millward, 2015; Moore, 2019, p. 57; Sewell, 2015; Valier & Bivins, 2002).

Similar patterns of marginalisation are playing out today. For instance, programmes to care for the elderly and vulnerable have once again relied on the mobilisation of hundreds of thousands of volunteers (to undertake phone calls, food deliveries and other tasks), whilst the continued reliance on racialised labour in key worker roles – combined with the persistent effects of structural violence – have meant that BAME communities are dying disproportionately of COVID-19. Moreover, though at the time of writing (May 2020) we have thankfully yet to see Government funding decisions forcing NHS staff to choose who might receive life-saving respiration and who misses out, the existence of standardised (yet culturally loaded) technologies for weighing the costs of different interventions against quality and quantity of life are of concern to those whose lives are often constructed as of “lesser” value (on the history of these technologies: Armstrong *et al.*, 2007; MacKillop & Sheard, 2018). Indeed, concerns among disability rights groups, and other organisations and communities, have been (legitimately) heightened by ethical discussions regarding “brutal” decisions on treatment that might be required soon ( British Medical Association, 2020), and by suspicions that the biopolitical calculations of loss embedded in conceptions of “herd immunity” ( Hinchliffe, 2020) are still informing government and NICE policy (despite utterances to the contrary).

Questions of who the NHS is willing and able to wait for, who does this waiting and how the continuation of some lives rather than others are prioritised in the temporality of crisis, have thus long been full of tensions and paradoxes. At present, prioritisation in risk minimisation – of stricter measures for some groups to prevent infection – does not seem to carry over to clinical decision-making where “value” is judged on different terms.

## Conclusion

As Lachenal & Thomas (2020) have suggested – also in conversation with Rosenberg (1989) – the coronavirus pandemic might be best considered to be an historically novel event, and historians should not rush to fit its ongoing devastation within any previously recognised frame. We can see this to some extent with the NHS. Previous outbreaks of infectious disease have strained the post-war British health services, albeit not in the same way as COVID-19. Struggles here often related to difficulties procuring sufficient vaccine material, or to coping with queues for vaccinations when demand spiked ( Millward, 2019, pp. 114–46). Moreover, efforts at containment were often local, reflecting the existence of local public health structures. By contrast, the current crisis is unprecedented in the NHS era, both in term of its scope and the national quarantine measures imposed.

Nonetheless, whilst trying to learn “lessons” from previous epidemics might be a problematic mission, it is nonetheless reasonable to place contemporary reactions in relation to longer-term trends in order to understand both where they have come from, and the particularities of local and national forms. From the preceding review, for instance, it is clear to see how the policy response to contain and delay has been framed within longer-term anxieties about health service demand and under-funding, decades-old frameworks of risk and long-held cultural and political values which have placed racialised, older and more vulnerable publics in a place of ambiguity. Themes of waiting, endurance and existential threat – how the NHS has been considered to historically (and biographically) wait for us, how we must now wait for the NHS if citizens and institutions are to endure, and whose existence might be threatened regardless from their ambiguous status as “vulnerable” – appear consistently throughout the history of the service. Equally, it is notable how governance strategies have sought to harness deep-rooted cultural expressions of attachment for the NHS in order to support its more individualising appeals for self-isolation and social distancing. For researchers, then, epidemics like COVID-19 not only show the socially and culturally novel – such as the new forms of sociality configured with technological change – but also how the present is continuously shaped by values and practices with long antecedents. This suggests that in order to gain better traction on the temporalities of current strategies of epidemic management – the constructions of urgency, priority, containment, delay and projection – we need to have a grasp on the historical structures and values shaping approaches to disease control.

## Data availability

All data underlying the results are available as part of the article and no additional source data are required.

## Author information

Martin D. Moore is a Research Fellow in the Wellcome Centre for Cultures and Environments of Health at the University of Exeter. He is author of *Managing Diabetes, Managing Medicine: Chronic Disease and Clinical Bureaucracy in Post-War Britain*, (Manchester: Manchester University Press, 2019) and co-editor – with Professor Mark Jackson – of *Balancing the Self: Medicine, Politics, and the Regulation of Health in the Twentieth Century*, (Manchester: Manchester University Press, 2020). His current research concerns histories of time, waiting and care in post-war British general practice.
