# Supplementary material for: Historicising “containment and delay”: COVID-19, the NHS and high-risk patients
Source: Wellcome Open Res. 2020 Jun 10;5:130. [Version 1] doi: 10.12688/wellcomeopenres.15962.1 (PMC7348669; doi:10.12688/wellcomeopenres.15962.1)
Supplement: Peer review history

# Peer review: Reviewer response for version 1

**DOI:** 10.21956/wellcomeopenres.17506.r39298

**Published:** 2020-07-09

**Authors:** Alex Mold

**Affiliations:** 1 Centre for History in Public Health, London School of Hygiene and Tropical Medicine, London, UK

This is a fascinating article that uses the recent history of the NHS to explore the response to COVID-19 and vice-versa. The notions of time and waiting are successfully deployed to highlight and explain some of the key facets of how the UK government and the NHS have dealt with the epidemic. The article is well-written, clearly structured and draws on a wealth of historical research. The parallels and disjunctures with the past are insightful. The argument that time is both a problem to be dealt with in relation to COVID-19 and a (partial) solution to the dangers of the NHS being overwhelmed is convincing, although the focus on time and waiting is not always maintained throughout the article. Here are some suggestions for how this theme could be developed further.

**The meaning/s of ‘delay’**

 The various ways in which ‘delay’ has functioned both in the response to COVID-19 and within the recent history of the NHS could be expanded on. In the context of COVID-19, ‘delay’ was deployed as a tactic to slow the spread of disease at the population level, but some of the other aspects of ‘delay’ and its histories and meanings could also be underscored. ‘Delay’ implies putting something off that is eventually realised, but did/does it also operate as a way to stop people from seeking or obtaining treatment altogether? To what extent did delay and waiting function as tools of demand management? You seem to imply this in the section on GPs, but this could be brought out more explicitly.

 On the other hand, ‘delay’ clearly has some negative consequences for individuals and for the NHS more broadly, with some patients waiting ‘too long’ before seeking out medical attention. It was striking, for instance, how this was an issue even during the peak of the COVID-19 epidemic, with doctors and other health officials encouraging patients with urgent medical needs to continue to come forward. ‘Delay’ thus seems to have various, sometimes conflicting, meanings and applications.

**The interaction between value, risk and waiting**

 It is clear that the elderly and other ‘at risk’ groups are marginalised, both within the response to the COVID-19 epidemic and in the longer-running history of the NHS, but can this be characterised as a form of waiting? Or is it something else? Are the most at risk supposed to wait? What are they waiting for? Or are they being discouraged/prevented from accessing treatment at all?

 If this is a form of waiting, then what role does time, and how it is valued, play in determining who should wait and who should not? For instance, treatment for some curative cancer patients continued throughout the epidemic, whereas treatment for some palliative patients was ‘paused’. What does this tell us about time and how it is valued? Is it quantity or quality of time? Or is it the utility to which time might be put? Whose time matters, and whose does not?

**Time and the ‘dramaturgic’ nature of epidemics**

 You draw on Rosenberg’s classic essays on the nature of epidemics to frame your article. I wonder if there is scope to explore these in a little more depth. By describing epidemics as ‘dramaturgic events’, Rosenberg was also saying something about the nature of time and how it functions within an epidemic: i.e. that there is a beginning, middle and end. Time is linear. But might it also be circular, or at least move backwards as well as forwards? In the case of COVID-19, some countries and regions that thought they had suppressed the virus are now experiencing localised outbreaks or even the beginning of a ‘second wave’. The progression through Rosenberg’s acts is not necessarily clear and unidirectional. Your article has lots of nice examples of how history repeats itself – how might this shed further light on time and COVID-19?

 Rosenberg also argues that epidemics end with a whimper, not a bang. How might waiting and time function in the aftermath of COVID-19? There is much discussion of increased waiting times for NHS treatment – what can the history of waiting tell us about how this might be managed? Or is it unprecedented?

 Overall, this is an excellent article. There is some scope to expand and deepen the analysis on the themes of time and waiting, but the piece stands perfectly well as it is.

Is the work clearly and accurately presented and does it cite the current literature?

Yes

If applicable, is the statistical analysis and its interpretation appropriate?

Not applicable

Are all the source data underlying the results available to ensure full reproducibility?

No source data required

Is the study design appropriate and is the work technically sound?

Yes

Are the conclusions drawn adequately supported by the results?

Yes

Are sufficient details of methods and analysis provided to allow replication by others?

Yes

Reviewer Expertise:

History of public health

I confirm that I have read this submission and believe that I have an appropriate level of expertise to confirm that it is of an acceptable scientific standard.

# Peer review: Reviewer response for version 1

**DOI:** 10.21956/wellcomeopenres.17506.r39057

**Published:** 2020-06-16

**Authors:** Matthew Smith

**Affiliations:** 1 Centre for the Social History of Health and Healthcare, University of Strathclyde, Glasgow, UK

This article neatly demonstrates how the response to the current pandemic is best understood by understanding the broader history of the NHS and the factors that have shaped it over the years. It cites a great deal of relevant secondary material as well as relevant media and medical literature. Its overall argument focuses on the theme of 'time', but this theme gets a little lost during the third section of the article, as other (relevant) themes, such as race and ethnicity, come into focus. Although I understand why 'time' is being emphasised, strengthening the discussion of these themes and others might expand the importance of the article, especially with reference to the way in which pandemics illuminate 'social value and institutional practice'. I focus on three themes that could be examined in more depth.

**Time**

 I wondered if there would be space to discuss how the pandemic highlights the paradox of time and waiting when it comes to public healthcare and the NHS. Medicine often requires urgency (for instance in terms of some cancer diagnoses), but patience can also be important (waiting for the body to heal itself). We have been waiting for life to return to normal, but this time of waiting has also given us time for reflection and reconsideration of everything from the role of nature and the importance of family and friends to the structure of the economy and welfare systems. Perhaps this should apply to thinking about the NHS as well. I also wondered if more could be written about the 'delay' strategy, which seems to have only delayed the UK's suffering and economic recovery. 

 Overall, the theme of time just gets a little lost towards the end so if that is really the key point of the article, the focus on it should remain a little more consistent.

**Racial and Ethnic Minorities**

 This theme emerges later on, but seems to be quite important, particularly due to the recent Black Lives Matter protests, Brexit and the future of staffing the NHS. I wondered about whether the double burden of BAME health workers during this crisis will make a difference in improving race relations throughout society and if there is any evidence of this happening historically. 

**The Vulnerability of the NHS**

 The NHS has long been a political football, but it has also been affected by demographic, technological and labour market trends. Although the recent focus has been on preventing Covid19 infections, more generally there has been a lack of focus on preventing chronic health conditions, especially mental illness. This has been highlighted during lockdown. The crisis has demonstrated a societal willingness not to let vulnerable patients fall victim to this infection, but has it convinced us that more progressive policies could play a role in reducing the chronic disease burden and making us more capable of dealing with unforeseen crises such as this one? We tend to put these changes off (we are willing to wait for change) when we probably shouldn't be. 

 Overall, these suggestions are simply that - an attempt to deepen some of the arguments already made and perhaps tie together the them of 'time' a little more tighter. It's a well-written and valuable piece already.

Is the work clearly and accurately presented and does it cite the current literature?

Yes

If applicable, is the statistical analysis and its interpretation appropriate?

Not applicable

Are all the source data underlying the results available to ensure full reproducibility?

Yes

Is the study design appropriate and is the work technically sound?

Yes

Are the conclusions drawn adequately supported by the results?

Yes

Are sufficient details of methods and analysis provided to allow replication by others?

Yes

Reviewer Expertise:

History of health and medicine

I confirm that I have read this submission and believe that I have an appropriate level of expertise to confirm that it is of an acceptable scientific standard.
